# Effectiveness of physical and cognitive-behavioural intervention programmes for chronic musculoskeletal pain in adults: A systematic review and meta-analysis of randomised controlled trials

**DOI:** 10.1371/journal.pone.0223367

**Published:** 2019-10-10

**Authors:** Joyce Oi Suet Cheng, Sheung-Tak Cheng

**Affiliations:** 1 Norfolk and Norwich University Hospital NHS Foundation Trust, Norwich, United Kingdom; 2 Department of Health and Physical Education, The Education University of Hong Kong, Hong Kong, China; 3 Department of Clinical Psychology, Norwich Medical School, University of East Anglia, Norwich, United Kingdom; Ospedale Sant’Antonio, ITALY

## Abstract

This systematic review and meta-analysis aimed to examine the effects of physical exercise cum cognitive-behavioural therapy (CBT) on alleviating pain intensity, functional disabilities, and mood/mental symptoms in those suffering with chronic musculoskeletal pain. MEDLINE, EMBASE, PubMEd, PsycINFO and CINAHL were searched to identify relevant randomised controlled trials from inception to 31 December 2018. The inclusion criteria were: (a) adults ≥18 years old with chronic musculoskeletal pain ≥3 months, (b) randomised controlled design, (c) a treatment arm consisting of physical intervention and CBT combined, (d) the comparison arm being waitlist, usual care or other non-pharmacological interventions such as physical exercise or CBT alone, and (e) outcomes including pain intensity, pain-related functional disabilities (primary outcomes), or mood/mental symptoms (secondary outcome). The exclusion criteria were: (a) the presence of comorbid mental illnesses other than depression and anxiety and (b) non-English publication. The search resulted in 1696 records and 18 articles were selected for review. Results varied greatly across studies, with most studies reporting null or small effects but a few studies reporting very large effects up to 2-year follow-up. Pooled effect sizes (Hedges’ *g*) were ~1.00 for pain intensity and functional disability, but no effect was found for mood/mental symptoms. The effects were mainly driven by several studies reporting unusually large differences between the exercise cum CBT intervention and exercise alone. When these outliers were removed, the effect on pain intensity disappeared at post-intervention while a weak effect (g = 0.21) favouring the combined intervention remained at follow-up assessment. More consistent effects were observed for functional disability, though the effects were small (*g* = 0.26 and 0.37 at post-intervention and follow-up respectively). More importantly, the value of adding CBT to exercise interventions is questionable, as consistent benefits were not seen. The clinical implications and directions for future research are discussed.

## Introduction

Chronic musculoskeletal pain is the most common type of chronic pain suffered in older age groups [1], with a higher prevalence in women and lower income groups [2]. It is defined as persistent or recurrent pain for at least three months that arises as part of a disease process, such as inflammation secondary to infection, autoimmune disease or metabolic aetiology, or direct structural changes affecting bone, joints, muscle or soft tissue [3]. The World Health Organisation classification of musculoskeletal disorders categorises the diseases into five groups: inflammatory rheumatic diseases; osteoporosis and other bone diseases; osteoarthritis and related conditions; soft tissue periarticular disorders; and back pain. Irrespective of aetiology, individuals affected with chronic pain often suffer from increased muscle tension and intense pain affecting life activities, sleep disturbance, fatigue, and mood disturbance (anxiety and depression), which can inevitably contribute to increased job absence [1].

Chronic pain can be very difficult to manage because of its complex natural history, unclear aetiology and inadequate control despite advances in pharmacological treatment. In view of this, non-pharmacological interventions constitute the mainstay methods for the management of chronic musculoskeletal pain, with exercise therapies and cognitive-behavioural therapy (CBT) amongst recommended treatments [4].

Exercise, regardless of the type, is recommended for the management of patients with chronic musculoskeletal pain, while frequency has been shown to be more essential than the length or intensity of the physical activity [5]. A meta-analysis conducted by Searle et al. [6] analysed the effects of exercise interventions for the treatment of chronic low back pain. Pooling the results across 45 trials covering 4462 participants, they found a beneficial effect for strength/resistance and coordination/stabilisation type of exercise programmes with a small effect in terms of lowering pain severity. Another meta-analysis by Bertozzi et al. [7], covering seven studies with 664 participants, studied the effect of therapeutic exercise on the management of pain and disability in individuals with chronic nonspecific neck pain. They found that therapeutic exercise had medium effects on pain in the short- and intermediate-term.

Chronic pain is not simply a physical problem and is often associated with diverse psychological factors [8,9]. Research shows dysfunctional beliefs and feared expectations, and consequently avoidance behaviour, act as barriers to change. Chronic pain patients may become preoccupied with false beliefs and expectations that are catastrophic in nature, revolving around ideas such as loss of independent living, vulnerability, rapid and progressive pathology, and morbidity [10]. It is therefore not surprising that CBT has been investigated as a treatment of choice for people with chronic pain. Richmond et al. [11] reviewed the effectiveness of cognitive-behavioural approaches in improving disability, pain, quality of life or work disability for those affected by low back pain. Pooling results from 23 studies with a total of 3359 participants, they found that CBT had a small effect on alleviating disability and pain intensity, in comparison to waitlist control or usual care, while having a small to moderate effect on reducing pain and disability when compared with active treatment (after removing an outlier). Another review by Knoerl et al. [12] examined 35 studies regarding the effect of CBT on chronic pain, with the majority of the study population affected by back/neck pain. They found that CBT was effective for reducing pain intensity in 43% of the trials, though only eight of the included trials studied pain as a primary outcome. Their review also showed that online delivery methods were effective for individuals with fibromyalgia, back/neck pain and chronic pain of mixed aetiology. This was further supported by a meta-analysis (11 studies) by Macea and colleagues, with the pooled results favouring a small effect of web-based CBT for patients with chronic pain (mostly back pain and osteoarthritis), in comparison to waitlist control [13].

Although physical exercise and CBT have both received support, there is an argument for combining both approaches in the management of chronic pain. Patients only receiving physical treatment may attempt to enhance their aerobic capacity, muscle strength and endurance, but maladaptive beliefs and avoidance behaviour that may exist concurrently, may limit their commitment. On the other hand, those receiving only CBT may be willing to increase their activity level but their physical ability may prevent this. Thus, combining physical exercise and CBT may show greater effects on the individual by reconstructing adaptive beliefs to underpin positive health behaviours and restoring functional ability through increased fitness.

Despite the promise of blending physical exercise with CBT, there has been an absence of a review of the efficacy of such interventions. To fill this gap in the literature and to inform practice, we conducted a systematic review and meta-analysis of randomised controlled trials on the effects of such combined interventions, focusing on studies of persons with chronic musculoskeletal pain. Pain intensity and pain-related functional disability are the primary outcomes whereas mood or mental symptoms constitute the secondary outcome. The main purpose was to examine the performance of physical exercise cum CBT relative to nil treatment, waitlist, usual care, exercise alone, or CBT alone as control.

In addition, this review also examined the performance of physical exercise alone and CBT alone if the studies selected also included these treatment arms. Although this was not our primary aim, such comparisons were attempted because understanding how exercise or CBT performed in these studies would be useful for making sense of the findings concerning the intervention with the two components merged. Meta-analysis, however, were not be performed for this part of the review as the studies were not representative of the corresponding literature.

## Methods

### Protocol and registration

The review was registered with PROSPERO (identifier #98918). The protocol is available as online supporting information (S1 Protocol). The review was conducted in accordance with PRISMA guidelines (S1 Checklist). Ethics approval was not required for review studies by the authors’ institutions.

### Eligibility criteria

Published articles describing randomised controlled trials involving physical exercise and cognitive-behavioural programmes for individuals with chronic musculoskeletal pain were included. The inclusion criteria were: (a) adults ≥ 18 years old with chronic musculoskeletal pain for at least three months, (b) study using randomised controlled design, (c) a treatment arm consisting of physical intervention and CBT programmes (those involving cognitive restructuring) combined, (d) the comparison arm being nil treatment, waitlist, usual care or other non-pharmacological interventions such as physical exercise or CBT alone, and (e) outcomes including pain intensity, pain-related functional disabilities or mood/mental symptoms (using any validated data collection tools). The exclusion criteria were: (a) the presence of comorbid mental illnesses other than depression and anxiety (as diagnosed using any recognised diagnostic criteria) and (b) non-English publication.

### Information sources and search

We searched MEDLINE, EMBASE, PubMEd, PsycINFO and CINAHL Full Text to identify randomised controlled studies from inception to end of 2018 using the keywords TI/AB = pain, TI/AB = exercise or “physical activity” or physiotherapy, TI/AB = “cognitive behavioural” or “cognitive behavioral”, and TI/AB = program* or trial* or intervention*. No additional terms for outcomes was included to ensure the identification of all studies involving physical intervention and CBT programmes. We obtained additional articles by cross-referencing review articles and searching manually through reference lists of primary studies which met the inclusion criteria.

### Study selection

Titles and abstracts of studies retrieved were screened independently by the two authors to identify suitable studies that met the inclusion criteria. The full text of the potentially eligible studies were then assessed. Reasons for excluding studies were recorded and any disagreement between the two authors were resolved through discussion. Where there was insufficient information to determine eligibility, study authors were contacted and supplementary information was requested.

### Data collection process

Data from included studies were extracted using a standard (hard copy) form by the first author and checked by the second author. Study setting, participant demographics, methodology, recruitment, duration, treatment characteristics, length of follow-up, outcomes, tools used to measure outcomes, and information for risk-of-bias assessment were recorded. Means, *SD*s and sample size per treatment arm and time point were copied onto a spreadsheet. Where necessary, mean and *SD* were estimated from median and range of values [14]. Authors of studies were contacted where necessary to provide the data if not available from the article itself.

### Risk of bias

Risk of bias was assessed at the study level using the Cochrane risk-of-bias tool [15]. The two authors assessed the risk of bias independently across the seven domains of the tool for each study: random sequence generation; allocation concealment; blinding of participants and personnel; blinding of outcome assessment; incomplete outcome data; selective reporting; and other sources of bias. We rated each item as being at “low risk,” “unclear risk” or “high risk” of bias. Initial differences between the two raters were small and were resolved through discussion. We classified the overall risk of bias as low if all domains were at low risk of bias, as high if at least one of the domains was rated high risk, or as unclear if at least one domain was at unclear risk of bias. Results for individual studies and across studies are reported through tabular and graphical representation respectively. The bias judgements were used to interpret the strength of the evidence from the review when drawing conclusions.

### Summary measures

The primary outcomes include pain intensity and pain-related disabilities (i.e., the extent to which pain interferers with daily activities), whereas the secondary outcome is mood/mental state, such as depressive and anxiety symptoms. More description of these measures can be found in the Results section.

### Synthesis of results

A narrative synthesis of the findings from the included studies was conducted, structured around the type of intervention, target population characteristics, type of outcome and intervention content. For effect size estimate, we report Hedges’ *g* with correction for the baseline difference between groups. Effect sizes based on raw and marginal means were both considered for the narrative synthesis. In small samples with *N* < 50, correction for upward bias was applied. To aid interpretation, we unified the scoring so that a positive *g* always meant an effect favouring the intervention, and vice versa. As a rule of thumb, *g* values of 0.20, 0.50 and 0.80 represent small, medium and large effects respectively.

As mentioned before, although our objective was to examine the effects of the exercise cum CBT intervention, we would also review the effects of exercise- and CBT-alone interventions. Nevertheless, because there were very few studies of exercise- and CBT-alone interventions in this pool, and because these studies were not representative of the entire literature on these interventions, we did not conduct meta-analysis for them. As a result, meta-analysis was conducted for the comparison between the combined intervention and control conditions only.

For the meta-analysis, only *g*s based on raw (unadjusted) means/*SD*s were included. Three studies without such information were hence excluded, leaving 15 studies of which the results were pooled. To take into account heterogeneity of study results, random effects models were computed to yield average effect sizes, with standard errors adjusted using weights derived from the inverse-variance method [16]. When the same outcome was measured by more than one instruments in a study, the effects were averaged within the study before being subject to meta-analysis. Heterogeneity was indexed by Cochrane’s *Q* and *I*^2^ [17]. The dispersion of effects was displayed using forest plots.

We conducted two sets of meta-analysis using Stata 15.1 (StataCorp, College Station, Texas, US), one for outcomes at post-intervention and the other for outcomes at follow-up. For the latter, we took the last assessment up to 12 months after the end of intervention. To make the results more meaningful and to reduce potential heterogeneity, each set of meta-analysis was further subdivided to show results of comparison with three different types of control conditions—exercise only, CBT only, and nonspecific controls (including nil treatment, waitlist, usual care, and treatment-as-usual other than exercise and CBT). Note that the term “nonspecific control” was adopted for the sake of convenience only, without necessarily implying an absence of specific elements in the control condition. For example, usual care could involve specific services for helping pain patients.

### Publication bias

Contour-enhanced funnel plots were created and Egger’s tests [18] were used to examine asymmetry as a representation of small-study effect (i.e., whether studies with smaller samples tended to yield significant effects and get published). The Egger’s test was conducted separately for pain intensity and functional disability, but not for mood/mental symptoms as the number of studies were too small for detecting asymmetry. Given the small number of studies making it more difficult to detect asymmetry, we followed Egger et al.’s [18] recommendation to adopt *p* < 0.10 as suggesting the presence of small-study effect.

## Results

### Study selection

**Fig 1:**
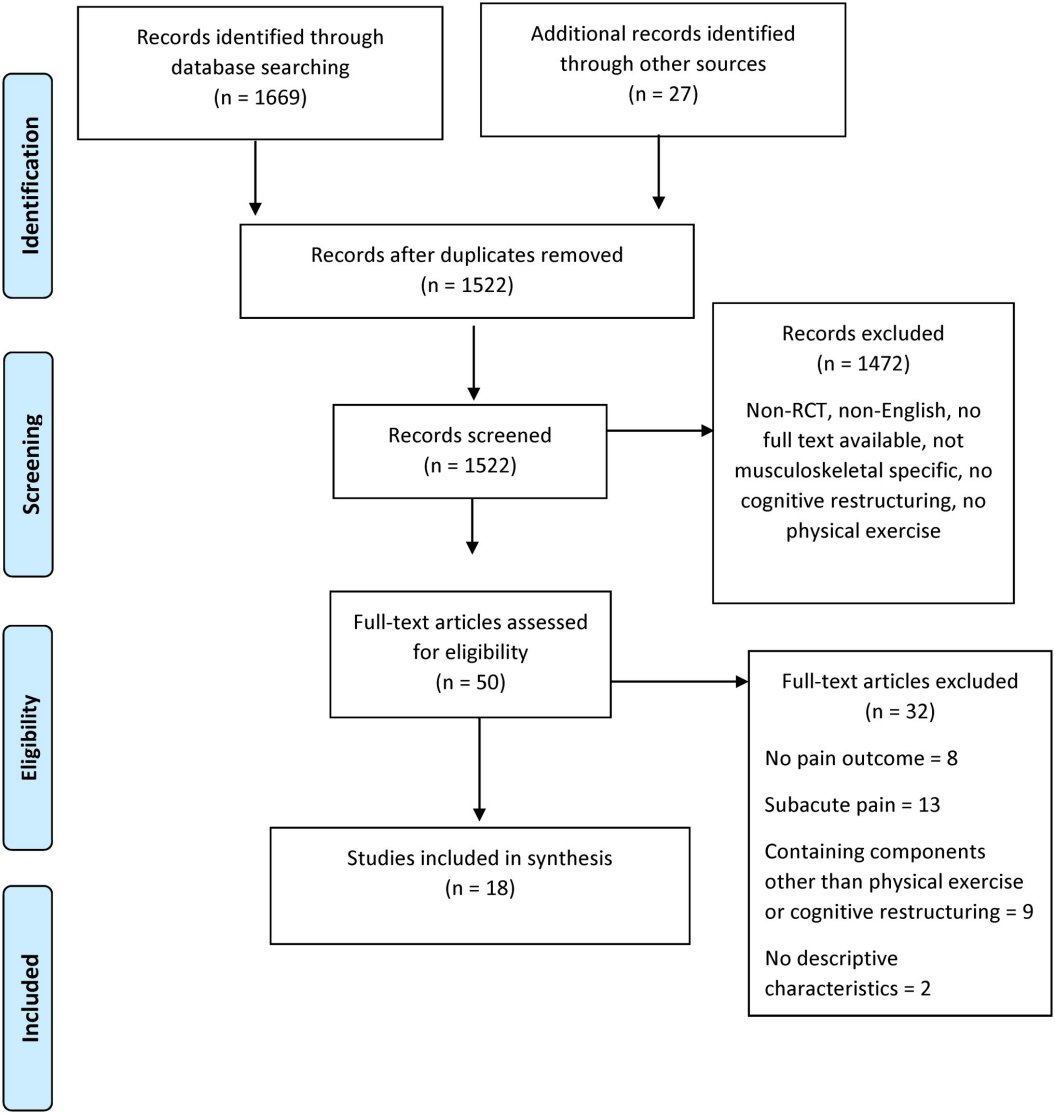
Flow chart of literature search.

The search resulted in 1669 records across the five databases. Twenty-seven additional records were identified from screening reference lists of previously published systematic reviews and included trials. After removal of duplicates, titles and abstracts of 1522 unique records were screened. We screened 50 full text articles and identified 18 randomised controlled trials to be included in this review, including one trial [19] for which the necessary information for calculating *d* could not be obtained from the authors. Five of these studies were conducted by Monticone and colleagues [20–24] on patients with chronic neck or low back pain; there was no indication that the patients in these studies overlapped and therefore they were treated as independent studies. The flow of the literature search is shown in Fig 1.

### Study characteristics

Details of the included studies are shown in Table 1. The 18 studies included 2391 participants with a mean age ranging from 37.3–63.4 years across studies. The geographical distribution of the studies were: Australia (*n* = 2), Finland (*n* = 1), Hong Kong (*n* = 1), Italy (*n* = 5), Netherlands (*n* = 2), Pakistan (*n* = 1), Singapore (*n* = 1), Spain (*n* = 1), Sweden (*n* = 1) and United Kingdom (*n* = 3). The range of musculoskeletal conditions included chronic (nonspecific) low back pain (*n* = 11), chronic neck pain (*n* = 3), chronic musculoskeletal pain (*n* = 1), osteoarthritis (*n* = 1), chronic widespread pain (*n* = 1) and fibromyalgia (*n* = 1). Intervention duration was mostly 1–3 months, with some interventions lasting as long as 6–12 months, while the length of follow-up varied from one month to two years after the intervention. Six out of 18 studies delivered their interventions in individual sessions, while seven studies performed their interventions in groups (but were mostly tailored to the individual) and one study used a combination of both. The intervention format could not be determined for four studies as no details were provided.

**Table 1:** Summary of studies included in the review.

Author, year	Condition	*n* (completing), mean age	Follow-up assessment	Control group, *n* (completing)	Intervention group, *n* (completing)	Treatment characteristics; individual/group; duration	Outcome measures	Effects	
								Post	Follow-up
*Bennell et al., 2016 [25]	Osteoarthritis	222 (186), 63.4	Post, 20 weeks, 40 weeks	PE alone, 75 (61)	CBT alone, 74 (61)	Individual: 10 sessions over 12 weeks	Pain intensity *Visual Analogue Scale*	*ns*	*ns*
							Functional disabilities *Western Ontario and McMaster Universities Osteoarthritis Index–Function subscale*	0.63	*ns*
							Mood and mental state *Depression*, *Anxiety Stress Scales*		
							*Depression subscale*	*ns*	0.50 (40 weeks)
							*Anxiety subscale*	*ns*	*ns*
					PE cum CBT, 73 (64)		Pain intensity *Visual Analogue Scale*	0.43	0.24 (40 weeks)
							Functional disabilities *Western Ontario and McMaster Universities Osteoarthritis Index–Function subscale*	0.70	0.47 (40 weeks)
							Mood and mental state *Depression*, *Anxiety Stress Scales*		
							*Depression subscale*	*ns*	0.56 (20 weeks)
							*Anxiety subscale*	*ns*	*ns*
				CBT alone, 74 (61)	PE cum CBT, 73 (64)		Pain intensity *Visual Analogue Scale*	0.49	0.50 (20 weeks)
							Functional disabilities *Western Ontario and McMaster Universities Osteoarthritis Index–Function subscale*	1.18	0.78 (20 weeks) 0.80 (40 weeks)
							Mood and mental state *Depression*, *Anxiety Stress Scales*		
							*Depression subscale*	*ns*	*ns*
							*Anxiety subscale*	*ns*	*ns*
Devasahayam et al., 2014 [19]	Chronic nonspecific low back pain	28 (15), 53.5	Post	PE alone, 14 (6)	PE cum CBT, 14 (9)	Group: Once / week for 4 weeks	Pain intensity *Verbal Numerical Pain Scale*	**+**^Δ^	NA
							Functional disabilities *Roland Morris Disability Questionnaire*	**+**^Δ^	NA
*Johansson et al., 1998 [26]	Chronic musculo-skeletal pain	42 (36), 43.5	Post, 1 month	Waitlist, 21 (19)	PE cum CBT, 21 (17)	Group (but individually tailored exercises): 5 sessions over 4 weeks	Pain intensity *Visual Analogue Scale*	*ns*	*ns*
							Functional disabilities *Multidimensional Pain Inventory–‘activity’ subscale*		
							*Household chores*	*ns*	*ns*
							*Outdoor work*	*ns*	*ns*
							*Activities away from home*	*ns*	*ns*
							*Social activities*	0.65	0.74
							*General activity level*	*ns*	0.66
*Kaapa et al., 2006 [27]	Chronic nonspecific low back pain	120 (95), 46.*ns*	Post 6 months, 12 months, 24 months	PE alone, 61 (46)	PE cum CBT, 59 (49)	Group (but individually tailored exercises): 70 hours over 8 weeks	Pain intensity *Numerical Rating Scale*	0.33	*ns*
							Functional disabilities *Oswestry Disability Index*	0.41	0.54
							Mood and mental state *Depression Scale*	0.37	*ns*
*Khan et al., 2014 [28]	Chronic low back pain	54 (54), 39.6	Post	PE alone, 27 (27)	PE cum CBT, 27 (27)	Not stated; 3 times / week for 12 weeks	Pain intensity *Visual Analogue Scale*	1.57	NA
							Functional disabilities *Roland Morris Disability Questionnaire*	2.29	NA
Lambeek et al., 2010 [29]	Chronic low back pain	134 (117), 46.1	Post, 3 months, 6 months, 9 months	Usual care, 68 (59)	PE cum CBT, 66 (58)	Not stated; 26 sessions within 12 weeks	Pain intensity *Visual Analogue Scale*	*ns*	*ns*
							Functional disabilities *Roland Morris Disability Questionnaire*	*ns*	0.62 (9 months)
*Lee et al., 2013 [30]	Chronic low back pain	47 (47), 37.3	Post	PE alone, 23 (23)	PE cum CBT, 24 (24)	Not stated; 11 sessions	Pain intensity *Numerical Pain Rating Scale*	*ns*	NA
							Functional disabilities *Roland Morris Disability Questionnaire*	*ns*	NA
*Macedo et al., 2012 [31]	Chronic nonspecific low back pain	172 (155), 49.2	Post, 2 months, 6 months, 12 months	PE alone, 86 (75)	PE cum CBT, 86 (80)	Individual; 14 sessions over 10 months	Pain intensity *Numerical Rating Scale*	*ns*	*ns*
							Functional disabilities *Roland Morris Disability Questionnaire*	*ns*	*ns*
*Martin et al., 2014 [32]	Fibromyalgia	153 (110); 50.2	20 weeks	Treatment as usual (pharma-cological treatment), 71 (56)	PE cum CBT (together with treatment as usual), 82 (54)	Not stated; 12 sessions in 6 weeks	Pain intensity *Fibromyalgia Impact Questionnaire–pain severity item*	NA	0.46
							Functional disabilities *Fibromyalgia Impact Questionnaire–total*	NA	0.46
							Mood and mental state *The Hospital Anxiety and Depression Scale*	NA	*ns*
*McBeth et al., 2012 [33]	Chronic widespread pain	442 (338), 56.2	Post, 3 months	Usual care, 109 (98)	PE alone, 109 (99)	Individual: At least twice / week with 6 instructor-led monthly appointments	Pain intensity and disability *Chronic Pain Grade Questionnaire*	*ns*	*ns*
							Functional disabilities *Chalder Fatigue Score*	*ns*	*ns*
							Mood and mental state		
							*General Health Questionnaire*	*ns*	*ns*
							*SF-36 Mental Component*	*ns*	*ns*
					CBT alone, 112 (91)	Individual: 7 weekly sessions, 1 session at 3 months and 1 session at 6 months	Pain intensity and disability *Chronic Pain Grade Questionnaire*	*ns*	*ns*
							Functional disabilities *Chalder Fatigue Score*	*ns*	*ns*
							Mood and mental state		
							*General Health Questionnaire*	*ns*	*ns*
							*SF-36 Mental Component*	*ns*	*ns*
					PE cum CBT, 112 (102)	As above combined	Pain intensity and disability *Chronic Pain Grade Questionnaire*	*ns*	*ns*
							Functional disabilities *Chalder Fatigue Score*	0.49	0.34
							Mood and mental state		
							*General Health Questionnaire*	*ns*	*ns*
							*SF-36 Mental Component*	*ns*	*ns*
				PE alone, 109 (99)	PE cum CBT, 112 (102)	As above combined	Pain intensity and disability *Chronic Pain Grade Questionnaire*	*ns*	*ns*
							Functional disabilities *Chalder Fatigue Score*	0.40	*ns*
							Mood and mental state		
							*General Health Questionnaire*	*ns*	*ns*
							*SF-36 Mental Component*	*ns*	*ns*
				CBT alone, 112 (91)	PE cum CBT, 112 (102)	As above combined	Pain intensity and disability *Chronic Pain Grade Questionnaire*	*ns*	*ns*
							Functional disabilities *Chalder Fatigue Score*	0.27	*ns*
							Mood and mental state		
							*General Health Questionnaire*	*ns*	*ns*
							*SF-36 Mental Component*	*ns*	*ns*
*Moffett et al., 1999 [34]	Chronic low back pain	187 (171), 42.*ns*	Post, 5 months, 11 months	Usual care, 98 (88)	PE cum CBT, 89 (83)	Group: 8 times over 4 weeks	Functional disabilities *Roland Morris Disability Questionnaire*	*ns*	0.23 (5 months) 0.36 (11 months)
Monticone et al., 2012 [20]	Chronic neck pain	80 (75), 49.6	Post, 12 months	PE alone, 40 (35)	PE cum CBT, 40 (40)	Individual: 12 sessions over 2–3 months	Pain intensity *Numerical Rating scale*	*ns*	*ns*
							Functional disabilities *Neck Pain and Disability Scale*	*ns*	*ns*
*Monticone et al., 2013 [21]	Chronic low back pain	90 (90), 49.3	Post, 12 months	PE alone, 45 (45)	PE cum CBT, 45 (45)	Individual: Once / week for 5 weeks then monthly for 1 year of CBT; twice / week for 5 weeks of PE	Pain intensity *Numerical Rating Scale*	3.29	3.97
							Functional disabilities *Roland Morris Disability Questionnaire*	3.41	3.40
*Monticone et al., 2014 [22]	Chronic low back pain	20 (20), 57.75	Post, 3 months	PE alone, 10 (10)	PE cum CBT, 10 (10)	Individual: Once / week for 8 weeks CBT, twice / week for 8 weeks of PE	Pain intensity *Numerical Rating Scale*	*ns*	*ns*
							Functional disabilities *Oswestry Disability Index*	*ns*	2.06
*Monticone et al., 2016 [23]	Chronic low back pain	150 (129), 53.5	Post, 12 months, 24 months	PE alone, 75 (64)	PE cum CBT, 75 (65)	Group (but individually tailored exercises): Once / week for 5 weeks CBT; twice / week for 5 weeks of PE	Pain intensity *Numerical Rating Scale*	2.05	1.27 (12 months)
									1.55 (24 months)
							Functional disabilities *Oswestry Disability Index*	2.34	3.53 (12 months)
									3.57 (24 months)
*Monticone et al., 2017 [24]	Chronic neck pain	170 (155), 52.9	Post, 12 months	PE alone, 85 (77)	PE cum CBT, 85 (78)	Group: Once / week for 10 weeks each for CBT and PE– 20 hours in total	Pain intensity *Numerical Rating Scale*	2.79	3.06
							Functional disabilities *Neck Disability Index*	2.24	2.84
*^#^Smeets et al., 2006 [35]	Chronic low back pain	223 (212), 41.6	Post	Waitlist, 51 (50)	PE alone, 53 (52)	Group (but individually tailored exercises): 3 times / week for 10 weeks	Pain intensity *Visual Analogue Scale* *McGill Pain Questionnaire—Pain Rating Index*	0.46 *ns*	NA NA
							Functional disabilities *Roland Morris Disability Questionnaire*	0.52	NA
							Mood and mental state *Beck Depression Inventory*	0.37	NA
					CBT alone, 58 (55)	Both individual and group sessions: 3 times / week initially then once / week for 10 weeks	Pain intensity *Visual Analogue Scale* *McGill Pain Questionnaire—Pain Rating Index*	0.79 *ns*	NA NA
							Functional disabilities *Roland Morris Disability Questionnaire*	0.65	NA
							Mood and mental state *Beck Depression Inventory*	*ns*	NA
					PE cum CBT, 61 (55)	As above combined	Pain intensity *Visual Analogue Scale* *McGill Pain Questionnaire—Pain Rating Index*	*ns* *ns*	NA NA
							Functional disabilities *Roland Morris Disability Questionnaire*	0.54	NA
							Mood and mental state *Beck Depression Inventory*	*ns*	NA
				PE alone, 53 (52)	PE cum CBT, 61 (55)	As above combined	Pain intensity *Visual Analogue Scale* *McGill Pain Questionnaire—Pain Rating Index*	*ns* *ns*	NA NA
							Functional disabilities *Roland Morris Disability Questionnaire*	*ns*	NA
							Mood and mental state *Beck Depression Inventory*	-0.39	NA
				CBT alone, 58 (55)	PE cum CBT, 61 (55)	As above combined	Pain intensity *Visual Analogue Scale* *McGill Pain Questionnaire—Pain Rating Index*	-0.39 *ns*	NA NA
							Functional disabilities *Roland Morris Disability Questionnaire*	*ns*	NA
							Mood and mental state *Beck Depression Inventory*	*ns*	NA
*Thompson et al., 2016 [36]	Chronic neck pain	57 (40), 47.5	5 months	PE alone, 28 (17)	PE cum CBT, 29 (23)	Group: 4 weeks (frequency unclear)	Pain intensity *Numerical Pain Rating Scale*	NA	*ns*
							Functional disabilities *Northwick Park Neck Pain Questionnaire*	NA	*ns*

*Note*. PE = physical exercise, CBT = cognitive-behavioural therapy, post = post-intervention, *ns* = nonsignificant, NA = not applicable (i.e., not tested). Where an effect was significant, Cohen’s *d* is displayed. A positive *d* means an effect favouring the intervention, or vice versa.

^Δ^Effect favouring intervention but effect size could not be calculated because necessary data are unavailable.

*Studies that were included in meta-analysis.

^#^For the measure of pain intensity, only the Visual Analogue Scale was included in meta-analysis as the required data for the McGill Pain Questionnaire were not available.

### Outcome measures

Pain intensity was assessed by Numerical Rating Scales [37]– 9 studies, Visual Analogue Scales [38]– 4 studies, Verbal Numerical Pain Scale [39]– 1 study, Fibromyalgia Impact Questionnaire (pain severity item) [40]– 1 study, and McGill Pain Questionnaire: Pain Rating Index study [41]– 1 study.

Functional disabilities were assessed by the Roland Morris Disability Questionnaire [42]– 8 studies, Oswestry Disability Index [43]– 3 studies, Neck Disability Index [44]– 1 study, Neck Pain and Disability Scale [45]– 1 study, the Western Ontario and McMaster Universities Osteoarthritis Index: Function subscale [46]– 1 study, the Multidimensional Pain Inventory: Activity subscale [47]– 1 study, Fibromyalgia Impact Questionnaire total [40]– 1 study, Chalder Fatigue Score [48]– 1 study, and Northwick Park Neck Pain Questionnaire [49]– 1 study. One study used the Chronic Pain Grade Questionnaire to measure the severity of both pain intensity and disability [50].

Mood and mental symptoms were measured by the Depression Anxiety Stress Scales [51]– 1 study, the Hospital Anxiety and Depression Scale [52]– 1 study, the General Health Questionnaire [53]– 1 study, SF-36 Mental Component score [54]– 1 study, the Beck Depression Inventory [55]– 1 study, and the Depression Scale [56]– 1 study.

### Intervention effects

At post-intervention, 15 studies assessed pain intensity and/or functional disability while four assessed depressive and/or anxiety symptoms. At follow-up, 14 studies assessed pain intensity and/or functional disability and four studies assessed mood or mental symptoms. Before we review the performance of physical exercise cum CBT interventions, we first discuss the effects of exercise-alone and CBT-alone interventions in this selected group of studies.

#### Physical exercise alone

Physical exercise interventions involved aerobic training to enhance cardiorespiratory fitness and dynamic static strengthening exercises. Only two studies examined physical exercise alone as a treatment, comparing it to waitlist control [35] or usual care [33]. McBeth and colleagues [33] found no effect of physical exercise at post-intervention or 3-month follow-up on any measure of pain intensity, disability and mental health in patients with chronic widespread pain (a main feature of fibromyalgia). Smeets and colleagues [35], however, reported small to moderate effects on pain intensity (*g* = 0.46), functional disability (*g* = 0.52), and depressive symptoms (*g* = 0.37) in patients with chronic low back pain after 10 weeks of aerobic and dynamic state strengthening exercises.

#### CBT alone

CBT programmes usually involved pain education and training in cognitive and behavioural skills for coping with pain, identifying and challenging pain-related negative thoughts, and modifying fear of movement. These thought modifications were then encouraged to be integrated into their daily activities. Only three studies [25,33,35] evaluated the effects of CBT as a stand-alone intervention. Again, in patients with chronic widespread pain, no effect on pain intensity, disability, or mental health was found [33]. Smeets and colleagues [35] found that a 10-week CBT consisting of both individual and group sessions had moderate effects on reducing pain intensity (*g* = 0.79) and functional disability (*g* = 0.65), but surprisingly no effect on depressive symptoms. This study did not assess follow-up outcomes. In Bennell and colleagues’ study [25], CBT was found to have a small effect on functional disability at post-intervention (*g* = 0.63) and also a medium effect on depressive symptoms at 40-week follow-up (*g* = 0.50), when compared with *physical exercise alone*, but no effect on pain intensity whatsoever. It was noteworthy that the effect on depressive symptoms was limited to the 40-week follow-up while no effects were observed at post-intervention and 20-week follow-up. Moreover, the effect on functional disability was limited to post-intervention. Hence the effects were either short-lived or inconsistent over time. It was the only study that provided a direct comparison between CBT and exercise and showed the former to be somewhat superior. (Note that in Table 1, when there were multiple follow-up time points [column labelled “Follow-up assessment”] and at least one of the follow-up effects was significant, only the one[s] with significant effect is shown in order to streamline presentation. Likewise, when there were multiple follow-up assessments but none was significant, an overall “*ns*” is shown.) On the whole, there was some evidence of a small-to-moderate immediate effect on reducing functional disability but the effect on depressive symptoms was weak and uncertain.

#### Physical exercise cum CBT

**Fig 2:**
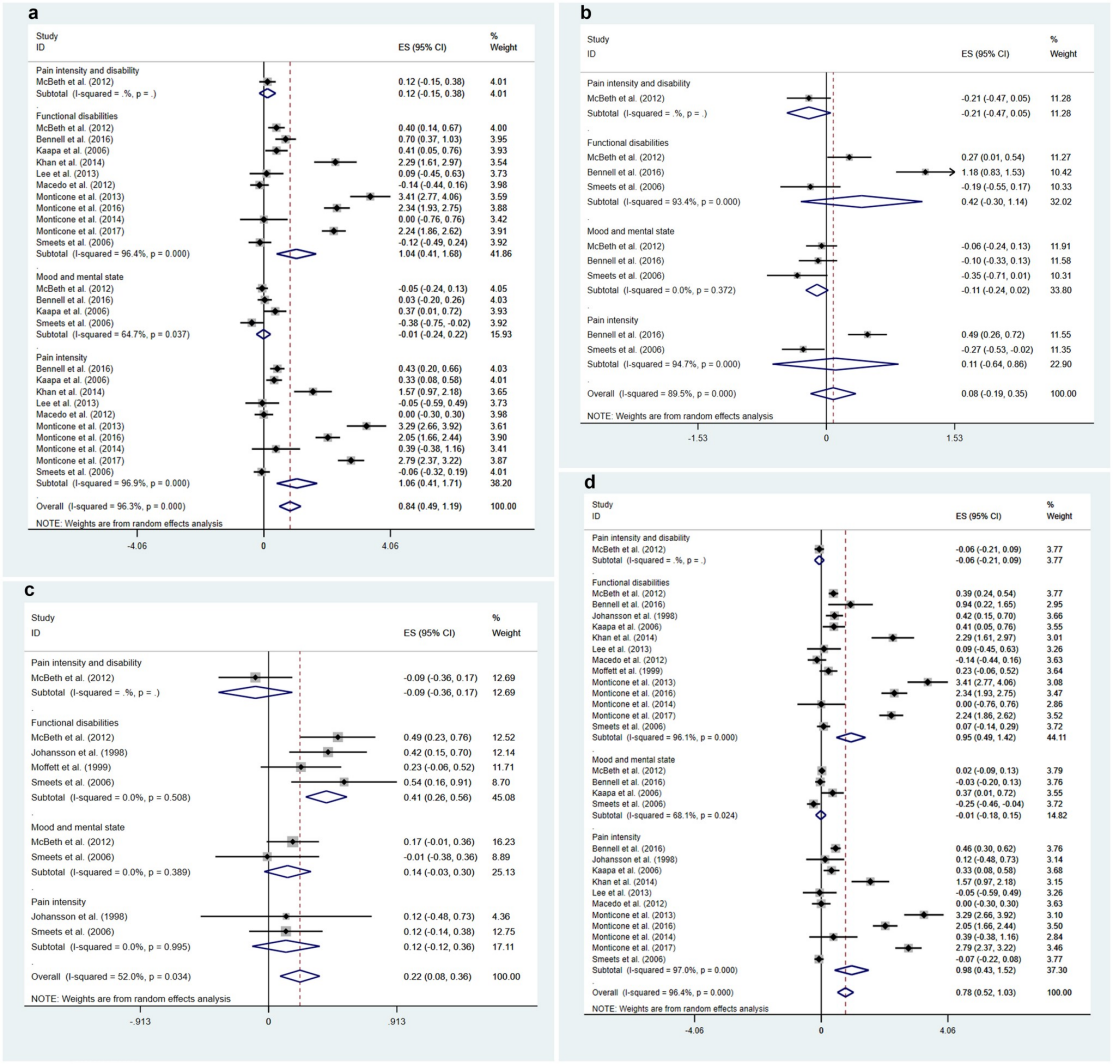
Forest plots for the effects of exercise cum cognitive-behavioural (CBT) intervention at post-intervention: (a) exercise alone as control, (b) CBT alone as control, (c) nonspecific control, and (d) all controls.

**Fig 3:**
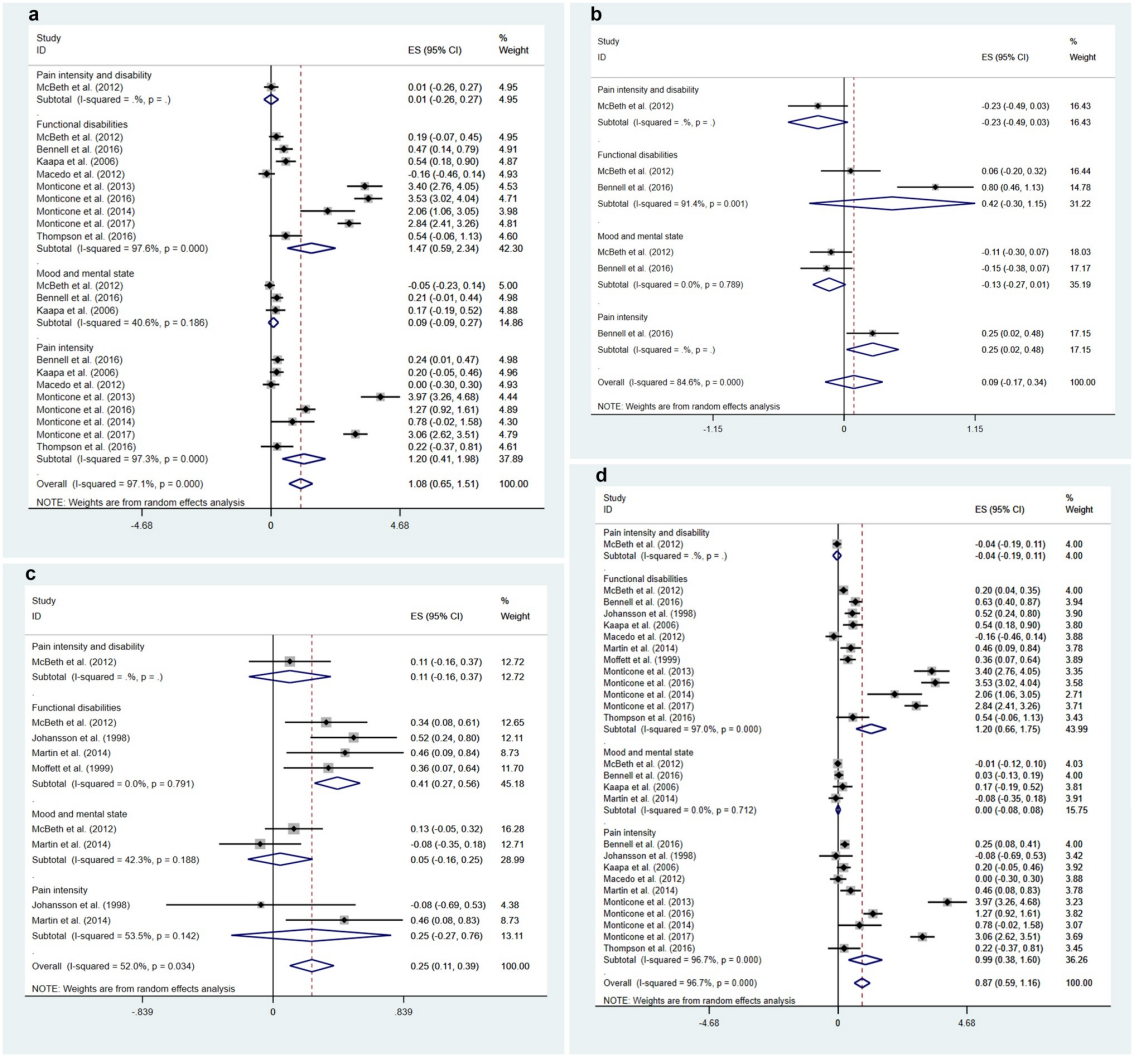
Forest plots for the effects of exercise cum cognitive-behavioural (CBT) intervention at follow-up: (a) exercise alone as control, (b) CBT alone as control, (c) nonspecific control, and (d) all controls.

We now come to the main review. All of the studies evaluated the effects of combining physical exercise and CBT, per our inclusion/exclusion criteria. Amongst the studies, two compared the combined intervention with waitlist control [26,35] and three with usual care [29,33,34]. One study compared the combined intervention with pharmacological therapy as treatment as usual [32]. Fourteen studies compared it to physical exercise alone [19–25,27,28,30,31,33,35,36], whereas only three used CBT alone [25,33,35] as a comparison group for the combined intervention. (The total count of studies exceeded 18 here as some studies offered more than one control/comparison group.) Thus, the majority of the studies attempted to assess the performance of the combined intervention against physical exercise alone. Typically in these cases, a CBT component was added to the exercise intervention which served both as a control and as a core part of the combined intervention. In other words, different from other studies having waitlist, usual care or treatment-as-usual as control, these studies were assessing whether adding CBT created additional benefits, beyond the effects of exercise alone. In the following, we provide an overview of the effects of such interventions on the three categories of outcome, namely pain intensity, functional disability, and mood and mental symptoms. Results of the meta-analysis are shown in Tables 2 and 3. Forest plots of the combined intervention’s effects, broken down by outcome categories and control conditions, are displayed in Figs 2 and 3. Note that the effect sizes shown in the forest plots may not be identical to those presented in Table 1 because of the selection of the last time point up to 12 months post-intervention (which may not appear in Table 1 if nonsignificant) and the within-study aggregation of effects across multiple measures for the same outcome category.

**Table 2:** Effects of exercise cum CBT intervention at post-intervention against different control conditions.

Variable and control condition	*k*	*n*	*g*	95% CI	*z*	*Q*	*I*^2^
Pain intensity							
Exercise alone	10	1085	1.06	0.42, 1.71	3.23***	287.96***	96.9%
CBT alone	2	266	0.11	-0.64, 0.86	0.29	19.01***	94.7%
Nonspecific control	2	148	0.12	-0.12, 0.36	1.01	0.00	0.0%
All control conditions^#^	11	1304	0.98	0.43, 1.52	3.50***	334.23***	97.0%
Functional disability							
Exercise alone	11	1306	1.04	0.41, 1.68	3.23***	275.80***	96.4%
CBT alone	3	490	0.42	-0.30, 1.14	1.14	30.39***	93.4%
Nonspecific control	4	448	0.41	0.26, 0.56	5.49***	2.33	0.0%
All control conditions^#^	13	1933	0.95	0.49, 1.42	4.00***	303.86***	96.1%
Mood and mental symptoms							
Exercise alone	4	603	-0.01	-0.24, 0.22	0.09	8.49*	64.7%
CBT alone	3	490	-0.11	-0.24, 0.02	1.63	1.98	0.0%
Nonspecific control	2	333	0.14	-0.03, 0.30	1.59	0.74	0.0%
All control conditions^#^	4	1007	-0.01	-0.18, 0.15	0.14	9.41*	68.1%

*Note*. *k* = number of studies; *n* = number of participants per analysis; CBT = cognitive-behavioural therapy.

^#^Some studies had more than one control condition; effects of the control conditions were averaged within studies to create an overall effect per study per outcome, following the recommendation of Higgins and Green [17].

*p < 0.05,

**p < 0.01,

***p < 0.001

**Table 3:** Effects of exercise cum CBT intervention at follow-up against different control conditions.

Variable and control condition	*k*	*n*	*g*	95% CI	*z*	*Q*	*I*^2^
Pain intensity							
Exercise alone	8	910	1.20	0.41, 1.98	2.99**	255.20***	97.3%
CBT alone	1	147	0.25	0.02, 0.48	2.14*	–	–
Nonspecific control	2	146	0.25	-0.27, 0.76	0.94	2.15	53.5%
All control conditions^#^	10	1130	0.99	0.38, 1.61	3.16***	269.26***	96.7%
Functional disability							
Exercise alone	9	1131	1.47	0.59, 2.34	3.29***	322.96***	97.6%
CBT alone	2	371	0.42	-0.30, 1.15	1.14	11.69***	91.4%
Nonspecific control	4	748	0.44	0.32, 0.57	7.07***	1.63	0.0%
All control conditions^#^	12	1759	1.20	0.66, 1.75	4.31***	369.62***	97.0%
Mood and mental symptoms							
Exercise alone	3	489	0.09	-0.09, 0.27	0.95	3.37	40.6%
CBT alone	2	371	-0.13	-0.27, 0.01	1.77	0.07	0.0%
Nonspecific control	2	408	0.05	-0.16, 0.25	0.43	1.73	42.3%
All control conditions^#^	4	894	0.00	-0.08, 0.09	0.07	1.37	0.0%

*Note*. *k* = number of studies; *n* = number of participants per analysis; CBT = cognitive-behavioural therapy. – = not applicable.

^#^Some studies had more than one control condition; effects of the control conditions were averaged within studies to create an overall effect per study per outcome, following the recommendation of Higgins and Green [17].

*p < 0.05,

**p < 0.01,

***p < 0.001

#### Pain intensity

Fourteen studies assessed pain intensity at post-intervention [19–26,26–31,35]. All but six studies found significant effects. Of the eight studies reporting significant effects, two [25,27] found generally small effects (*g* = 0.33–0.49) favouring the combined intervention over CBT- and exercise-only programmes, but one study [35] actually found an effect (*g* = -0.39) favouring CBT alone over the combined intervention. However, four others found surprisingly very large beneficial effects (*g* = 1.57–3.29) for the combined intervention for patients with low back pain and neck pain; except for one study [28], all were conducted by the same research group [21,23,24]. (Effect size estimate was not available for the Devasahayam et al. study [19].) Thus, there were substantial variations in study results, with some studies not showing an effect and a few with “outlying effects.” It was also noteworthy that most of the studies with waitlist or usual care control did not find an effect, whereas all but one significant effect favouring the combined intervention were obtained with exercise alone as control. This pattern was reflected in the meta-analysis of results at post-intervention (Table 2), showing a large and significant effect favouring the combined intervention over exercise alone (pooled *g* = 1.06 [95% CI: 0.42, 1.72]), but nonsignificant effects against CBT alone (pooled *g* = 0.11 [-0.64, 0.86]) or nonspecific control (pooled *g* = 0.12 [-0.12, 0.36]). This issue will be taken up again in Discussion, although the pattern cannot be easily explained.

One study [34] used patients’ diaries to score pain intensity but did not describe the scoring method. After excluding this study, a total of 12 studies reported follow-up outcomes on pain intensity [20–27,29,31,32,36], although not all reported post-intervention outcomes at the same time. Note that one of these studies [21] was listed as having 1-year follow-up outcomes, but in fact the CBT component of the combined intervention continued on a monthly basis after the conclusion of the main intervention; this needs to be taken into consideration when interpreting the results. Six of the included studies reported multiple follow-up time points. Of the 22 follow-up effects assessed, only seven effects reported by five studies were statistically significant. Two studies [25,32] reported small to medium effects (*g* = 0.24–0.50), mostly at ~5 months of follow-up, for patients with osteoarthritis and fibromyalgia, while again three studies by Monticone and colleagues [21,23,24] reported quite large effects (*g* = 1.27–3.97) up to 2-year follow-ups for patients with neck and low back pain. These effects were obtained with exercise alone [21,23–25], CBT alone [25], or pharmacological treatment [32] as control. Note that one study [33] analysed chronic pain grade, a measure combining pain intensity and disability, and did not find any effect at post-intervention or follow-up. (For the sake of thoroughness, this outcome also appears on the forest plots).

Meta-analytic results for pain intensity at follow-up (Table 3) showed a similar pattern to those at post-intervention, with a large effect against exercise-only programmes, pooled *g* = 1.20 (0.41, 1.98). However, there was a small effect when the combined intervention was compared with CBT alone (*g* = 0.25 [0.02, 0.48] based on one study only), but not when compared with nonspecific control (pooled *g* = 0.25 [-0.27, 0.76]). Thus, support for the combined intervention came primarily from the comparison with exercise-only programmes, while the results were rather mixed as evidenced by the degree of heterogeneity. Because of the large effects reported by some of these studies, the overall effect sizes, pooled across all control conditions, were *g* = 0.98 (0.43, 1.52) at post-intervention and 0.99 (0.38, 1.61) at follow-up (Tables 2 and 3).

#### Functional disability

All but two studies examined disability as a post-intervention outcome [19–26,26–31,33–35]. McBeth and colleagues [33] reported small effects (*g* = 0.27–0.49 against all three types of control conditions in patients with chronic widespread pain. Smeets and colleagues [35] found a moderate effect (*g* = 0.54) for low back pain patients when exercise cum CBT was compared with waitlist control, but no effect when it was compared with exercise- or CBT-alone programmes. Contrary to Smeets et al.’s results, Kaapa et al. [27] reported a small effect (*g* = 0.41) against exercise alone on patients with low back pain, whereas Bennell et al. [25] found large effects (*g* = 0.70 and 1.18) for the combined intervention for osteoarthritic patients, against exercise alone and CBT alone respectively. Using a waitlist control, Johansson and colleagues [26] conducted the only study in this pool in which different subdomains of functional ability were assessed, in patients with musculoskeletal pain. They found a positive moderate effect on social activity (*g* = 0.65) but not other types of activity. Additionally, four studies conducted by Khan et al. [28] and Monticone et al. [21,23,24] reported large effects for neck and low back pain patients (*g* = 2.24–3.41), all with exercise alone as control. On the contrary, five other studies did not find a significant effect on functional disability for exercise cum CBT at post-intervention [20,29–31,34]. On the whole, there was some evidence, though varied and inconsistent, that physical exercise cum CBT reduced functional disability at post-intervention, compared with waitlist, usual care, or exercise alone. Indeed, meta-analysis (Table 2) showed a large post-intervention effect against exercise alone for the combined intervention (pooled *g* = 1.04 [0.41, 1.68]), a small effect against nonspecific control (pooled *g* = 0.42 [0.29, 0.54]), but a nonsignificant effect against CBT alone (pooled *g* = 0.42 [-0.30, 1.14]).

All but four studies reported follow-up outcomes [20–27,29–31,33,34,36]. Of the 30 effects assessed in 14 studies, 12 were significant. Johansson and colleagues [26] reported effects at 1-month follow-up (*g* = 0.34–0.85) that were larger than those at post-intervention. Lambeek et al. [29] reported a moderate effect (*g* = 0.62) against usual care at 9-month follow-up only, but not at two shorter follow-up intervals (as well as at post-intervention). Bennell and colleagues [25] found moderate effects up to 40-week follow-up (*d* ~0.50) against CBT alone, but not against the exercise-alone programme. Martin and colleagues [32] also found a small effect at 20-week follow-up (*g* = 0.45) against pharmacological treatment. And again, the studies by Monticone and colleagues [21–24] reported large effects up to 2-year follow-up (*g* = 1.71–5.49). Six other studies testing 13 follow-up effects did not find any significant effect whatsoever [20,27,31,33,36], including the study by Moffett et al. [34]. This latter study reported significant effects at follow-up using change scores but the effects were not significant after recalculation using our effect size formula.

Again, pooling the follow-up results across studies showed a large effect against exercise alone for the combined intervention (pooled *g* = 1.47 [0.59, 2.34]), a small effect when compared with nonspecific control (pooled *g* = 0.44 [0.32, 0.57]), but no effect when compared with CBT alone (pooled *g* = 0.42 [-0.30, 1.15]). On the whole, there were more consistent effects on functional disability than on pain intensity, and the overall effects (*g*) regardless of control condition (post-intervention: 1.20 [0.66, 1.75]; follow-up: 0.95 [0.49, 1.42]) were larger at post-intervention. The bulk of the evidence, again, came from the comparison with exercise-alone programmes, with substantial heterogeneity in the findings of these studies. Interestingly, studies that reported positive results at post-intervention also tended to find significant results at follow-up, and vice versa.

#### Mood and mental symptoms

Only five studies assessed mood or mental symptoms, including depressive symptoms, anxiety, general psychological distress (General Health Questionnaire) and mental health (SF-36 Mental Component) at post-intervention or follow-up [25,27,32,33,35]. Only two of these studies found partial support for the effect of the combined intervention. Bennell and colleagues measured depressive and anxiety symptoms but did not find any effect at post-intervention. At follow-up, there were no effects whatsoever with CBT alone as control, but when exercise alone was the control, significant effects were found for depressive symptoms (*g* = 0.37 at 20-week follow-up only) and anxiety symptoms (*g* = 0.24 at 40-week follow-up only). Another study which found an effect was the one by Smeets and colleagues [35], showing a small effect too (*g* = 0.33) against exercise alone, but not when the combined intervention was compared with CBT alone or waitlist control. In both of these studies, effects were obtained when the exercise-alone condition served as the comparison group. No effect was obtained whatsoever when CBT alone was the reference group, but also no effect was found when waitlist [35] or usual care [33] served as control. Another study evaluating the combined intervention against pharmacological treatment was also unable to obtain a treatment effect [32]. However, the data for this study is to be interpreted with care as the dosages of antidepressants and analgesia used were suboptimal for the management of depression.

All of the five studies were included in meta-analysis. There was no support for the combined intervention in terms of alleviating mood and mental symptoms, whether using exercise alone, CBT alone, nonspecific control, or any control condition as the reference.

### Adherence to treatment

Six studies mentioned explicitly the procedures to monitor treatment fidelity (i.e., that treatment conditions were delivered as planned) and adherence to treatment by participants [23,25,29,31,33,36]. Four studies described monitoring treatment fidelity but not participant compliance [20–22,24], whereas three studies had explicit procedures to check participant compliance but not treatment fidelity [19,30,35]. In five other studies, there was some mentioning of monitoring participant adherence but the procedure was not clear or questionable [21,22,24,28,32]. Three studies, however, did not provide any description of procedures to monitor treatment fidelity or participant compliance [26,27,34]. There did not appear to be any connection between whether these procedures were in place and whether significant effects were found. However where effects were not found, whether the treatment was implemented according to protocol or whether participants were following the treatments as recommended remained a question.

### Risk of bias ratings

**Fig 4:**
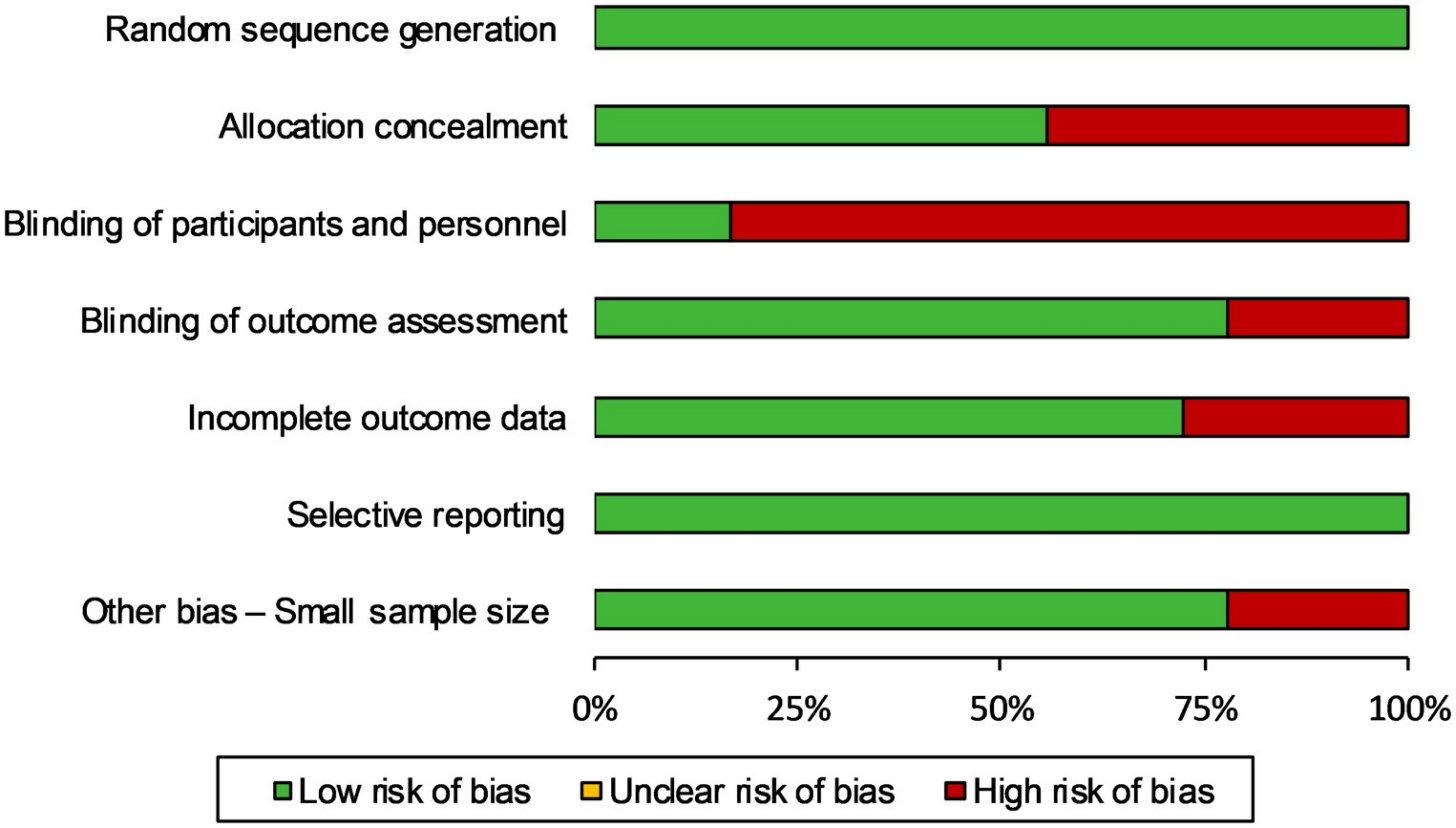
Distribution of risks of bias across studies.

The risk of bias in individual studies is shown in Table 4. Out of the seven domains assessed, only two domains were deemed low risk in all the studies (random sequence generation and selective reporting). As all studies were randomised controlled trials, random sequence generation was fulfilled. Allocation concealment was not performed in eight of the studies. Because of the nature of psychological and behavioural interventions, making it difficult to mask them, blinding of participants and personnel were generally poor. Nonetheless, blinding of outcome assessment was achieved successfully in 14 studies. The majority of studies used an intent-to-treat approach; several studies reported to have no attrition while others handled missing data by imputation or statistical modelling. All studies were deemed successful in preventing reporting bias. Small sample sizes were noted in 4 studies. The distribution of the levels of risks across studies is presented in Fig 4. Overall, all but one study was rated as having high risk of bias, where studies had at least one or more domains scoring “high risk.” None was rated as having unclear risk.

**Table 4:** Risk of bias across studies.

	Random sequence generation	Allocation concealment	Blinding of participants and personnel	Blinding of outcome assessment	Incomplete outcome data	Selective reporting	Other bias–small sample size
Bennell, 2016 [25]	+	+	+	+	+	+	+
Devasahayam, 2014 [19]	+	+	+	+	-	+	-
Johansson, 1998 [26]	+	-	-	-	-	+	-
Kaapa, 2006 [27]	+	+	-	-	+	+	+
Khan, 2014 [28]	+	-	-	-	+	+	+
Lambeek, 2010 [29]	+	+	-	+	+	+	+
Lee, 2013 [30]	+	-	+	+	+	+	-
Macedo, 2012 [31]	+	+	-	+	+	+	+
Martin, 2014 [32]	+	+	-	+	-	+	+
McBeth, 2012 [33]	+	-	-	+	-	+	+
Moffett, 1999 [34]	+	+	-	-	+	+	+
Monticone, 2012 [20]	+	-	-	+	+	+	+
Monticone, 2013 [21]	+	-	-	+	+	+	+
Monticone, 2014 [22]	+	-	-	+	+	+	-
Monticone, 2016 [23]	+	-	-	+	+	+	+
Monticone, 2017 [24]	+	+	-	+	+	+	+
Smeets, 2006 [35]	+	+	-	+	+	+	+
Thompson, 2016 [36]	+	+	-	+	-	+	+

*Note*: + = low risk of bias, ? = unclear risk of bias, – = high risk of bias.

### Publication bias

**Fig 5:**
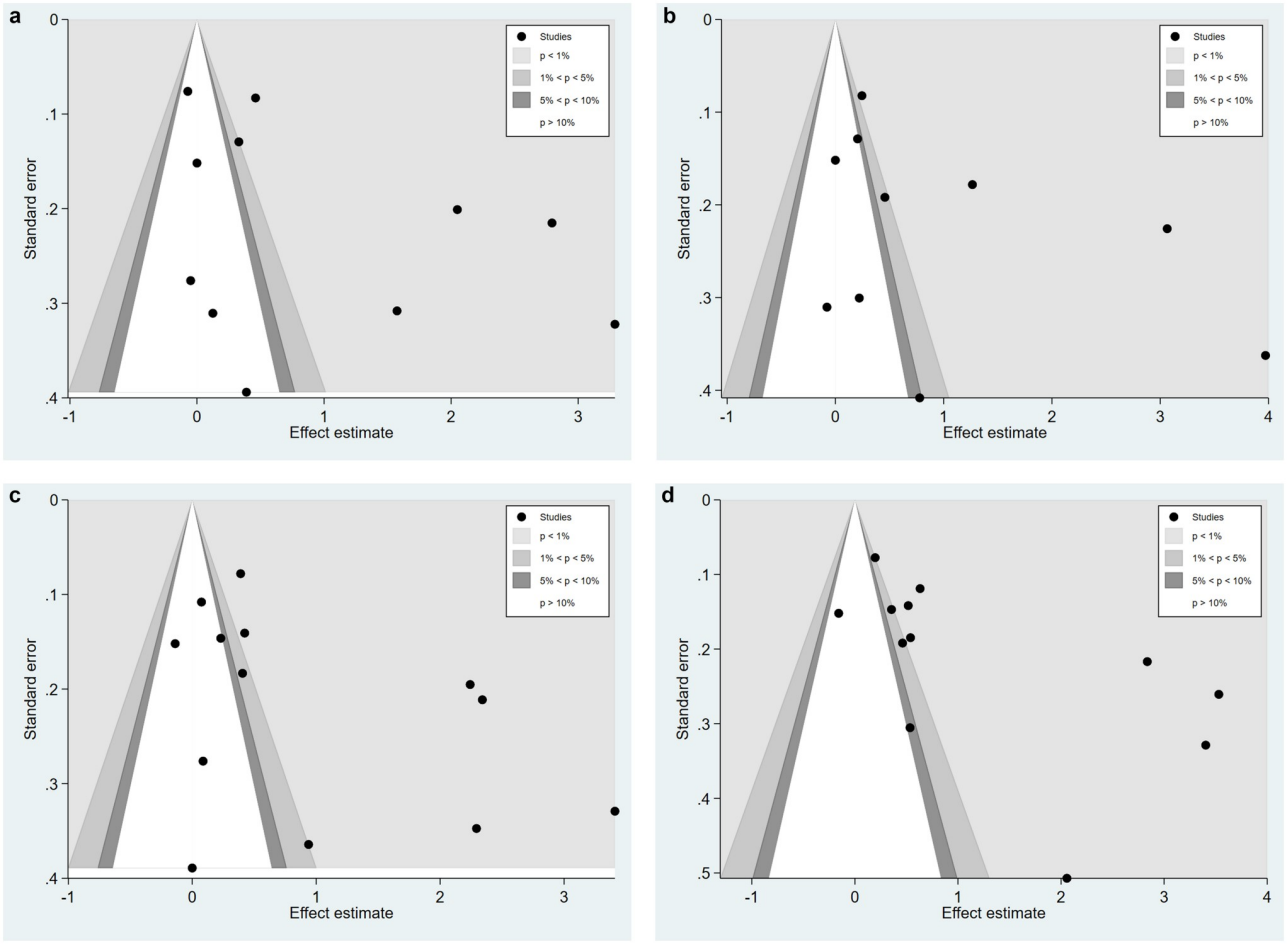
Funnel plots for (a) pain intensity at post-intervention, (b) pain intensity at follow-up, (c) functional disability at post-intervention, and (d) functional disability at follow-up, all control conditions.

Only the outcomes of pain intensity and functional disability had at least 10 studies for creating funnel plots. The funnel plots using all the studies available (i.e., any control condition) are displayed in Fig 5. Egger’s tests showed significant asymmetry at post-intervention (bias = 6.00, *t* = 2.01, *p* = 0.08) but not follow-up (bias = 5.58, *t* = 1.63, *p* = 0.14) for pain intensity. As for functional disability, there was asymmetry at both post-intervention (bias = 5.39, *t* = 1.95, *p* = 0.08) and follow-up (bias = 8.28, *t* = 2.74, *p* = 0.02). While the funnel plots were supposed to reveal whether small-study effects existed, the pattern actually suggested a bias toward large intervention effects being reported by studies with small to medium sample sizes.

### Sensitivity analysis

We attempted to examine outliers using several methods: (a) those outside of 95% CI, (b) those beyond |2 *SD*s| and (c) those beyond |3 *SD*s|. We decided that the last approach was the most appropriate one for this dataset as the former two led to too many outliers, although quite a number of outliers were still identified using the last method in an iterative fashion. For pain intensity at post-intervention, the number of outliers for the different control conditions were: 6 (exercise alone) and 8 (all controls). For functional disability at post-intervention, the number of outliers were: 8 (exercise alone) and 9 (all controls). For mood/mental symptoms at post-intervention, the number of outliers were: 2 (exercise alone), 1 (CBT alone), and 1 (all controls). For pain intensity at follow-up, the number of outliers were: 4 (exercise alone) and 7 (all controls). For functional disability at follow-up, the number of outliers were: 5 (exercise alone) and 6 (all controls). And for mood/mental symptoms at follow-up, there was 1 outlier when all studies, regardless of control condition, were analysed together.

After removing outliers at post-intervention, the effect sizes for pain intensity and functional disability, respectively, were *g* = 0.28 (0.07, 0.49) and 0.49 (0.31, 0.68) against exercise alone, and 0.42 (0.29, 0.56) and 0.37 (0.26, 0.49) against all controls. Likewise, when outliers at follow-up were removed, the effect sizes for pain intensity and functional disability, respectively, were *g* = 0.17 (0.03, 0.32) and 0.38 (0.20, 0.56) against exercise alone, and 0.23 (0.10, 0.37) and 0.52 (0.39, 0.65) against all controls. In other words, after the removal of outliers, the combined intervention’s effects on pain intensity and functional disability continued to be supported, although the magnitude of the effects was drastically reduced–only small effects found for pain intensity and small-to-medium effects for functional disability. The outliers for mood and mental symptoms did not alter conclusions after removal (i.e., no intervention effects whatsoever) and the detailed results will not be presented.

The above analyses had a potential problem. Because the effects reported by Khan et al. and Monticone et al. were so “off the chart,” their presence could shift the mean of the study effects to such an extent that studies finding no effects could be considered as outliers. We therefore attempted another set of sensitivity analysis by simply removing the five studies by Khan et al. and Monticone et al. [21–24,28]. The results were surprising. At post-intervention, the effect sizes for pain intensity and functional disability, respectively, became *g* = 0.16 (-0.06, 0.38) and 0.23 (-0.05, 0.51) against exercise alone, and 0.15 (-0.09, 0.40) and 0.26 (0.09, 0.43) against all controls. At follow-up, the effect sizes for pain intensity and functional disability, respectively, were *g* = 0.17 (0.03, 0.32) and 0.28 (0.01, 0.56) against exercise alone, and 0.21 (0.10, 0.32) and 0.37 (0.18, 0.56) against all controls. Hence, at post-intervention, only the effect on functional disability against all controls were significant; at follow-up, all the effects concerned remained significant but were diminished. In other words, the observed effects of the combined intervention were contributed mostly by two research groups who compared it to exercise-only programmes. When their studies were removed, no effects on the two primary outcomes were found at post-intervention, and only weak effects at follow-up assessments remained.

## Discussion

This review systematically analysed up-to-date evidence from 18 studies covering 2391 participants from 10 countries. To the best of the authors’ knowledge, it is the first to synthesise the effects of physical exercise cum CBT interventions on pain intensity, functional disability, and mood and mental symptoms in those suffering from chronic musculoskeletal disease. For a condition that is difficult to manage and often resistant to treatment, there is a vast amount of management options available with a lack of clarity on their efficacy. This review provides an overview of the combined efficacy of two widely used non-pharmacological options, namely physical exercise and CBT. It is assumed that the combined intervention helps to restore the physical condition of the patients, improve their skills to cope with pain, and encourage and empower them to take responsibility for the management of their own musculoskeletal pain. By helping patients modify their mistaken fears and beliefs, and thus adopt appropriate positive health-seeking behaviours, such interventions should be poised to support patients to contain the impact of pain on daily activities, thereby reducing functional disability and fostering independence. But does the literature support this proposition? Before the evidence is considered, a discussion of the potential impact of study bias is warranted.

### Risk of bias

Biases of design, which could affect how the evidence was weighed, were noted amongst the studies. The most common biases lied in the lack of allocation concealment and blinding of research participants and personnel. The latter problem is often unavoidable for psychosocial and behavioural interventions. Though not ideal, it was not considered a serious threat to the validity of these studies, especially when an equally credible treatment, such as exercise alone, was used as control. As only one study [26] has used a waitlist control as the only reference group for the exercise cum CBT intervention, we do not think the overall conclusion permissible from this pool of studies needs to be qualified further because of the existence of this bias.

As regards to the allocation concealment bias, eight studies were considered to be at high risk [26,28,30,33], over half of which belonged to the studies by Monticone et al. [20–23] and Khan et al. [28], which then led to another issue for consideration. It was not clear to what extent the lack of allocation concealment had to do with the range of effects reported by Monticone et al. and Khan et al., but given those effects being clear outliers, the existence of this bias suggests considerable caution when interpreting their findings. Khan et al. also did not blind outcome assessment, making it even more vulnerable to bias. These concerns are especially pertinent considering the fact that their studies had all used exercise alone as the reference group, thus constituting a disproportionate share of the evidence on the superiority of adding CBT to exercise interventions. With the potential bias of these studies taken into account, severe caution should be exercised when appraising the overall evidence concerning the relative performance of the combined intervention and exercise-alone programmes. These and similar issues will be revisited as the intervention effects are discussed in detail below.

### Effects of exercise cum CBT interventions

This review showed inconsistent effects of the combined intervention on pain intensity at post-intervention and follow-up time points. The majority of studies which assessed post-intervention pain intensity did not find an effect [20,22,25–29,31,33,35]. Similarly, half of the studies which reported follow-up outcomes found no effect [20,22,26,27,29,31,33], but when the number of assessments was taken into account for studies with multiple follow-up time points, the great majority of the evaluations did not provide support for the interventions.

The studies which found an effect reported moderate to large effects at post-intervention and small to large effects up to 2-year follow-up. Yet, it was noteworthy that most of these significant effects came from two research groups led by Khan and Monticone [21,23,24,28], contributing several very large effects to the pool. There was not a pattern suggesting a connection between sample size and whether significant findings were reported; many studies with quite large sample sizes powered to detect small effects did not actually find an effect. No pattern between other biases and study findings could be identified also. In fact, apart from the studies by Khan et al. and Monticone et al., only four studies [19,25,27,32] reported significant post-intervention or follow-up effects, out of a subset of 10 studies (after removing studies by Khan et al. and Monticone et al.). In fact, after removing these studies [21–24,28] from the pool, no overall intervention effect on pain intensity was found anymore, while only a weak effect remained at follow-up.

On the contrary, effects on functional disability were more often found, including studies where effects on pain intensity were absent. Most studies reported either post-intervention or follow-up effects that were generally small to medium in magnitude [19,25–27,29,32–35], except for those reported by Khan et al. [28] and Monticone et al. [21–24]. Several interventions which did not impact on pain intensity eased functional disability [22,26,29,33,35]. Note that one of these studies [26] had a number of methodological weaknesses, as evident in five of the seven Cochrane items being rated at high risk for this study (Table 4). Nevertheless, the conclusion remains unchanged if this study was to be removed from the pool. Moreover, this study was excluded from the meta-analysis due to the required information being unavailable and so its potential biases did not influence the pooled results. On the whole, there is moderate support for the interventions’ capability in improving daily activities *in spite of* pain. Even after removing the studies with unusually large effect sizes [21–24,28], small overall intervention effects on functional disability (all studies) were observed at post-intervention and follow-up. The effects appeared to be driven by comparisons between the combined intervention and nonspecific control, showing consistent effects on functional disability at post-intervention and follow-up (both *g*s > 0.40), despite having no effects on pain intensity.

A closer inspection of Table 1 suggests that when an effect on functional disability was reported in the absence of a simultaneous effect on pain intensity, the control group tended to be a waitlist condition or usual care [26,29,33,35], while physical exercise alone was the control in one study which was an outlier [22]. Given the preponderance of exercise alone serving as control in this batch of studies, the compelling conclusion is that the differential effects on functional disability (versus pain intensity) were *not* primarily driven by the superiority of the combined intervention over exercise alone.

In other words, while the effect on pain intensity was weak and inconsistent, if not for the outliers, the increased benefits on functional disability were *not* a result of adding CBT to exercise interventions either. Moreover, the combined intervention had no effect on mood and mental symptoms.

If adding CBT to exercise interventions yields no additional benefits (other than a few outliers), does it mean that CBT is not useful for patients with chronic pain? This issue is worthy of further consideration. The effectiveness of CBT has been established in those suffering from chronic nonspecific low back pain, according to a meta-analysis [11]. Short and long-term effects, though small to moderate in magnitude, were seen in the improvement of pain, functional disability and quality of life, when CBT was compared with guideline-based active treatment as well as usual care or waitlist. Another meta-analysis [57] assessing the effects of CBT on chronic pain (excluding headache and cancer pain) showed that it had moderate effects in improving pain intensity, mood symptoms and functional disability, as well as cognitive coping and appraisal (including catastrophising, i.e., a tendency to exaggerate and to ruminate about pain sensation and its effects) when compared with waitlist control. The effects were limited to pain intensity and catastrophising when CBT was compared with another active treatment.

In light of the established efficacy of CBT, the likely explanation for the relative lack of the effect of CBT, on top of physical exercise, is that the effects of CBT and physical exercise are more or less redundant. If this is true, then a possible reason is that the two types of treatment affect pain-related outcomes through common pathways. For example, pain catastrophising, a direct target of CBT, has also been found to be altered after physical exercise. The trial by Smeets and colleagues [35], as shown in Table 1, included four arms, namely, physical exercise alone, CBT alone, physical exercise cum CBT, and waitlist control. In addition to reporting on outcomes, they also conducted a series of analyses to see if changes in pain catastrophising and perceived internal control of pain from pre- to post-intervention mediated the intervention effects [58]. With waitlist as the reference group, they found that catastrophising, but not perceived control, mediated the effects on pain intensity and functional disability, *regardless of* the type of intervention. Furthermore, pain catastrophising mediated the improvement in depressive symptoms as well but only in those receiving the exercise-alone intervention. That is, even physical exercise was able to reduce catastrophising (although exactly how was not clear) which in turn explained the treatment effects on pain-related outcomes.

### Strengths

This study has several strengths. It is the first to review the effects of combining physical exercise and cognitive-behavioural restructuring amongst those suffering from chronic musculoskeletal pain. The studies together covered a large aggregate sample involving six musculoskeletal conditions and a wide age range, increasing the generalisability of the results to the chronic pain population in general. All of the studies used validated questionnaires to score subjective experiences. The outcomes observed are clinically important and relevant for researchers and practitioners concerned with the treatment of chronic musculoskeletal pain. Most studies reported on follow-up as well as post-intervention outcomes so that long-term effects of the interventions were available. This review also assessed whether fidelity and adherence to treatment were related to the outcomes. Risks of bias (including publication bias) and the effects of outlying studies were evaluated, with adjustments to conclusions being made accordingly.

### Limitations

Notwithstanding the strengths, the study has several limitations. First, in order to show how exercise cum CBT interventions perform in relation to different control conditions, analyses involving CBT-only and nonspecific control had only a few studies. It will be important to re-conduct such analyses when more studies are available so as to see whether the findings are replicated and to yield more reliable estimates of effect sizes. Second, only a few studies evaluated the effects of the combined intervention on mood and mental symptoms. More studies are needed not only to assess the effects of such combined interventions on these symptoms, but also to understand why the interventions have not been more effective. Third, the studies included participants with different diseases, which might contribute to heterogeneity of results.

Last but not least, many studies reported significant results at follow-up with intervals ranging from one month to nearly two years after the end of treatment. This begs the question of the factors that were responsible for sustaining the intervention effects over such long periods. For example, did the participants continue to follow the physical exercise regime and/or engage in cognitive restructuring on a regular basis, after the termination of treatment? No information was available from the studies to shed light on this important issue and to understand the mechanisms by which the large, long-term follow-up effects were produced in some studies. Future research should investigate the underlying factors that need to be incorporated into the design of intervention programmes in order to maximise their benefits to patients.

## Conclusion and future directions

In summary, judging from the largely inconsistent intervention effects as well as generally null results of the meta-analysis after removal of outliers, there is little evidence supporting the use of exercise cum CBT intervention for relieving pain intensity. However, a fair degree of support exists for its efficacy in reducing the impact of pain on everyday activities. The effect, a small one, was mostly limited to the comparison with control conditions such as waitlist and usual care. Yet, the value of adding CBT to exercise interventions is questionable, as evident from the fact that few differences were observed between such interventions and interventions consisting purely of physical exercise, other than a few studies with unusual results that were limited to two particular research sites.

Moreover, there was little evidence that interventions guided by CBT are better than physical exercise alone in improving mood. These findings beg the question of whether the extra manpower and cost to run the additional CBT component are warranted, in view of the marginal benefits they have over physical exercise alone. A caveat is that while adding CBT to exercise interventions may not be very worthwhile, CBT itself, when conducted independently, is an effective intervention for people with chronic pain as demonstrated in the literature.

In view of the limited efficacy and potential adverse effects of pharmacological treatment for chronic pain, more research is needed to understand how non-pharmacological interventions such as CBT and physical exercise should be utilised to help these patients. This may entail investigating person-level characteristics (e.g., psychological profile) that are associated with responsiveness to one type of treatment over another. Such research is needed to match individuals to treatment in order to maximise treatment gain. Research is also needed to understand the therapeutic processes involved in CBT and exercise interventions, and why the two types of intervention tend to have effects that are redundant. Furthermore, more research is needed to examine the effects of non-pharmacological interventions on psychological distress, given the prevalence of depression and anxiety symptoms in this population. Finally, implicit in the above arguments is the need to improve monitoring of treatment fidelity and participant compliance which are fundamental to the accurate assessment of treatments.

## Supporting information

S1 Checklist (DOCX)

S1 Protocol (DOCX)
