# Supplementary material for: Effectiveness of physical and cognitive-behavioural intervention programmes for chronic musculoskeletal pain in adults: A systematic review and meta-analysis of randomised controlled trials
Source: PLoS One. 2019 Oct 10;14(10):e0223367. doi: 10.1371/journal.pone.0223367 (PMC6786598; doi:10.1371/journal.pone.0223367)
Supplement: S1 Protocol — (DOCX) [file pone.0223367.s002.docx]

**A systematic review of the effectiveness of physical and cognitive-behavioral intervention programs for chronic musculoskeletal pain in adults**

**Study Protocol**

Joyce Oi Suet Cheng^1^, Sheung-Tak Cheng^2,3^

^1^Norfolk and Norwich University Hospital NHS Foundation Trust, UK

^2^Department of Health and Physical Education, The Education University of Hong Kong, Hong Kong

^3^Department of Clinical Psychology, Norwich Medical School, University of East Anglia, UK

*Study Question*

How effective are physical and cognitive-behavioral intervention programs in alleviating pain intensity, pain-related functional disabilities, and mood/mental symptoms in chronic musculoskeletal pain patients compared with nil treatment, usual care or other non-pharmacological interventions.

*Inclusion/Exclusion Criteria*

The inclusion criteria will be: (a) adults ≥ 18 years old with chronic musculoskeletal pain for more than 3 months, (b) study using randomised controlled design, (c) a treatment arm consisting of physical intervention and CBT programmes (those involving cognitive restructuring) combined, (d) the comparison arm being usual care or other non-pharmacological interventions such as physical exercise or CBT alone, and (e) outcomes including pain intensity, pain-related functional disabilities or depressive symptoms (using any validated data collection tools). The exclusion criteria will be: (a) the presence of comorbid mental illnesses other than depression and anxiety (as diagnosed using any recognised diagnostic criteria) and (b) non-English publication.

*Literature Search*

We will search the following five electronic bibliographic databases: MEDLINE, EMBASE, PubMed, PsycINFO, and CINAHL, and the reference lists of the eligible studies and review articles. The search strategy will include only terms relating to or describing the physical and cognitive-behavioral intervention programs for pain. The same search terms will be used with all bibliographic databases. The search will be limited to articles published in English. Studies published till the date the searches are run will be sought. The searches will be re-run just before the final analyses and further studies retrieved for inclusion. Sample search terms are: TI/AB = “cognitive behavioral”, TI/AB = program* or trial* or intervention*, TI/AB = exercise or “physical activity” or physiotherapy, and TI/AB =pain.

*Data Extraction*

Titles and/or abstracts of studies retrieved using the search strategy and those from additional sources will be screened independently by two review authors to identify studies that potentially meet the inclusion criteria outlined above. The full text of these potentially eligible studies will be retrieved and independently assessed for eligibility by two review team members. Any disagreement between them over the eligibility of particular studies will be resolved through discussion with a third reviewer.A standardized, pre-piloted form will be used to extract data from the included studies. Extracted information will include: study setting; study population and participant demographics and baseline characteristics; details of the intervention and control conditions; study methodology; recruitment and study completion rates; outcomes and times of measurement; suggested mechanisms of intervention action; information for assessment of the risk of bias. Two review authors will extract data independently, discrepancies will be identified and resolved through discussion (with a third author where necessary). Missing data will be requested from study authors.

*Risk of Bias Assessment*

The risk of bias will be assessed independently by two review authors using the Cochrane Collaborations’ risk of bias assessment tool. Study characteristics including randomization sequence generation, treatment allocation concealment, blinding, completeness of outcome data, selective outcome reporting, and other sources of bias will be considered. Disagreements between the review authors over the risk of bias in particular studies will be resolved by discussion, with involvement of a third review author where necessary.

*Acknowledgment and Delcaration of Interest*

The review is funded by Tai Hung Fai Charitable Foundation. The funding body plays no role in study conception, design, data synthesis, and data interpretation. We have no interest to declare.
